# Kidney Function in Patients With Neuromuscular Disease: Creatinine Versus Cystatin C

**DOI:** 10.3389/fneur.2021.688246

**Published:** 2021-09-24

**Authors:** Elles M. Screever, Jenny E. Kootstra-Ros, Joyce Doorn, Jellie A. Nieuwenhuis, Henk E. J. Meulenbelt, Wouter C. Meijers, Rudolf A. de Boer

**Affiliations:** ^1^Department of Cardiology, University Medical Center Groningen, Groningen, Netherlands; ^2^Department of Laboratory Medicine, University Medical Center Groningen, Groningen, Netherlands; ^3^Department of Pulmonary Disease, University Medical Center Groningen, Groningen, Netherlands; ^4^Department of Rehabilitation Medicine, University Medical Center Groningen, Groningen, Netherlands

**Keywords:** Cystatin C, creatinine, neuromuscular disorder, Duchenne muscular dystrophy, kidney function

## Abstract

**Background:** Accurate measurement of kidney function in patients with neuromuscular disorders is challenging. Cystatin C, a marker not influenced by skeletal muscle degradation, might be of clinical value in these patients.

**Methods:** We consecutively enrolled 39 patients with neuromuscular disorders. We investigated the association of the eGFR, based on plasma creatinine and Cystatin C, with clinical and biochemical variables associated with kidney function, namely age and galectin-3.

**Results:** Creatinine-based eGFR was 242 (±80) and Cystatin C-based eGFR was 110 (±23) mL/min/1.73 m^2^. Cystatin C-based eGFR was associated with age (β −0.63 *p* < 0.0001) and galectin-3 levels (β −0.43 *p* < 0.01), while creatinine-based eGFR was not (β −0.22 *p* = 0.20; β −0.28 *p* = 0.10). Sensitivity analyses in Duchenne and Becker patients revealed the same results: Cystatin C-based eGFR was associated with age (β −0.61 *p* < 0.01) and galectin-3 levels (β −0.43 *p* = 0.05), while creatinine-based eGFR was not (β −0.32 *p* = 0.13; β −0.34 *p* = 0.14).

**Conclusions:** These data indicate that estimation of renal function in patients with neuromuscular disorders cannot reliably be achieved with creatinine, while Cystatin C appears a reasonable alternative. Since a large proportion of patients with neuromuscular disorders develops heart failure, and requires heart failure medication, adequate monitoring of renal function is warranted.

## Introduction

Neuromuscular disorders comprise several diseases causing progressive weakness and disruption of muscle mass, which may eventually lead to respiratory (1) and cardiac complications (2, 3) and sudden death (4). Longer survival, due to improvement of therapy and early intervention, in combination with better treatment of cardiac complications, might contribute to renal dysfunction (5). Nowadays, renal failure is the third leading cause of death in this patient population. As shown by a Japanese longitudinal cause-of-death analysis, 14% of deceased patients with Duchenne Muscular Dystrophy (DMD) died from renal dysfunction (6). Creatinine levels are typically very low in patients with neuromuscular disorders as a result of muscle degradation and turnover. This makes the use of creatinine to asses estimated glomerular filtration rate (eGFR) a real limitation, which results in overestimation of kidney function (7, 8). Despite the latter, physicians generally use eGFR based upon plasma creatinine levels to predict kidney function.

Cystatin C, a cysteine protease inhibitor and an acknowledged biomarker for kidney function, might be a better fit in this specific patient population, as it is freely filtered by the glomerulus and not influenced by skeletal muscle degradation (9, 10). A case report on two muscular dystrophy patients showed that Cystatin C eGFR was in good agreement with renal clearance calculated by inulin, the gold standard for determination of GFR. Creatinine eGFR greatly overestimated renal function on the other hand (11). Therefore, we retrospectively studied the association of the eGFR, calculated by the Chronic Kidney Disease Epidemiology Collaboration (CKD-EPI) formula based on either plasma Cystatin C or creatinine levels, with clinical and biochemical parameters associated with renal function.

## Materials and Methods

### Study Population

The current study is a retrospective cohort study of patients with neuromuscular disorders that were screened for possible cardiac involvement or treated for established cardiac involvement, during routine outpatient care of the University Medical Center Groningen (UMCG), in Groningen, the Netherlands. In total 39 patients were included in this study. All patients were ≥18 years of age. The study protocol conforms to the ethical guidelines of the 1975 Declaration of Helsinki. Due to the retrospective nature, the Institutional Review Board (IRB) waived the need for informed consent.

Patient demographics, medical history, laboratory measurements, ventilation status, pulmonary function (by spirometry), left ventricular function (by echocardiography) and biochemical markers of renal function and cardiac function were collected.

### Biomarker Assays

Plasma creatinine levels were measured with the use of the Roche enzymatic creatinine assay on a Roche Modular/Cobas e602 platform (Roche Diagnostics, Mannheim, Germany), traceable to National Institute of Standards and Technology creatinine standard reference material (SRM 967) (12). Plasma Cystatin C concentrations were measured routinely with a immunoturbidimetric assay, on a Roche Cobas C502 platform (Roche Diagnostics, Mannheim, Germany), standardized to the International Federation of Clinical Chemistry (IFCC) Working Group for Standardization of Cystatin C (13). N-terminal B-type natriuretic peptide (NT-proBNP) levels were measured using a commercially available electrochemiluminescent sandwich immunoassay on a Roche Modular/Cobas e602 platform (Roche Diagnostics, Mannheim, Germany). High-sensitivity Troponin T (hs-TnT) levels were measured using a fifth-generation high-sensitivity immunoassay, on a Roche Modular/Cobas e602 platform (Roche Diagnostics, Mannheim, Germany). All tests were performed in Lithium Heparin plasma. Galectin-3 was measured with a chemiluminescent microparticle immunoassay (CMIA) using the Abbott ARCHITECT automated immunoassay analyzer (Abbott Park, IL, USA), in EDTA plasma.

### Estimates of Renal Function

Creatinine and Cystatin C levels were obtained from the same blood draw from all patients.

eGFR values were calculated for creatinine using the CKD-EPI equation (14):


$$\begin{array}{l} \text{eGFR}\left(\text{mL}/\text{min}/1.73{\text{m}}^{2}\right)=141\times \min{\left({\text{S}}_{\text{cr}}/\text{κ},1\right)}^{\text{α}}\\ \;\times \text{max}{\left({\text{S}}_{\text{cr}}/\text{κ},1\right)}^{-1.209}\times 0.993^{\text{age}}\\ \;\times 1.018\left[\text{if female}\right]\times 1.159\left[\text{if black}\right] \end{array}$$

S_cr_ (standardized creatinine) = μmol/L

κ = 0.7 if female, 0.9 if male

α = −0.329 if female, −0.411 if male

min = indicates the minimum of S_cr_/κ or 1

max = indicates the minimum of S_cr_/κ or 1

age = years

eGFR values were calculated for Cystatin C using the CKD-EPI Cystatin C equation (2012) (15):


$$\begin{array}{l} \text{eGFR}\left(\text{mL}/\text{min}/1.73{\text{m}}^{2}\right)=133\times \min{\left({\text{S}}_{\text{cys}}/0.8,1\right)}^{-0.499}\\ \;\times \text{max}{\left({\text{S}}_{\text{cys}}/0.8,1\right)}^{-1.328}\times 0.996^{\text{age}}\\ \;\times 0.932\left[\text{if female}\right] \end{array}$$

S_cys_ (standardized Cystatin C) = mg/L

min = indicates the minimum of S_cys_/0.8 or 1

max = indicates the maximum of S_cys_/0.8 or 1

age = years.

### Statistical Analysis

Continuous data are presented as means (±SD) if normally distributed and as medians (interquartile range [IQR]) if non-normally distributed. Categorical variables are presented as number (frequency). Biomarker levels were log transformed prior to analysis to obtain approximately normal distributions. Differences between two groups were analyzed with the use of the Student's *T*-test for normally distributed data, the Mann-Whitney *U* test for non-normally distributed data and the Spearman's chi square test for categorical variables. Linear regression analysis was performed to demonstrate the correlation between eGFR and either galectin-3 levels or age. All reported *p* values are two-tailed. A *p* < 0.05 was considered to indicate statistical significance. Analyses were performed with STATA software (version 16.0; Stata Corp, College Station, TX, USA).

## Results

### Patient Characteristics

Baseline characteristics of the 39 muscular dystrophy patients are presented in Table 1. The mean age of the study subjects at evaluation was 31 (±11) years, 82% were male and 59% were diagnosed with either Duchenne or Becker Muscular Dystrophy. Mean FEV1 was 2.0 (±1.4), mean FVC was 2.2 (±1.4), 46% of the patients needed respiratory support. Mean left ventricular ejection fraction (LVEF) was 46% (±12), 1 of the patients (3%) was treated with corticosteroids and 51% of the patients used either angiotensin-converting enzyme inhibitors (ACEi), angiotensin II receptor blockers (ARB) or mineralocorticoid receptor antagonists (MRA). Median creatinine value was 10 μmol/L [7–23] and for Cystatin C it was 0.81 mg/L [0.71–0.92]. Median calculated creatinine-based eGFR was 242 (±80) and for Cystatin C-based eGFR it was 110 (±23) mL/min/1.73 m^2^.

**Table 1:** Baseline characteristics of neuromuscular dystrophy patients.

**Characteristics**	**Total population (*n* = 39)**
Age at evaluation (y), mean (SD)	31 (11)
Male sex, *n* (%)	32 (82)
Body mass index (kg/m^2^), mean (SD)	23 (5)
**Muscular dystrophy**	
Duchenne muscular dystrophy, *n* (%)	20 (51)
Spinal muscular atrophy, *n* (%)	5 (13)
Becker muscular dystrophy, *n* (%)	3 (8)
Morbus steinert/Myotonic dystrophy type 1, *n* (%)	3 (8)
Metabolic myopathy, *n* (%)	1 (3)
Congenital myopathy, *n* (%)	4 (10)
Limb girdle muscular dystrophy, *n* (%)	3 (8)
**Respiratory status**	
FEV1 (L), mean (SD)	2.0 (1.4)
FVC (L), mean (SD)	2.2 (1.4)
No support, *n* (%)	21 (54)
Invasive ventilation, *n* (%)	9 (23)
Nocturnal support, *n* (%)	9 (23)
All day support, *n* (%)	9 (23)
Ejection fraction (%), mean (SD)	46 (12)
LVEF ≥ 50, *n* (%)	20 (56)
LVEF 40–49, *n* (%)	9 (25)
LVEF <40, *n* (%)	7 (19)
**Medication**, ***n*** **(%)**	
Corticosteroids	1 (3)
ACEi	18 (46)
ARB	2 (5)
MRA	3 (8)
**Laboratory measurements**	
Cystatin C (mg/L), median [IQR]	0.81 [0.71–0.92]
Creatinine (umol/L), median [IQR]	10 [7–23]
Cystatin C-based eGFR (mL/min/1.73 m^2^), mean (SD)	110 (23)
Creatinine-based eGFR (mL/min/1.73 m^2^), mean (SD)	242 (80)
NT-proBNP (ng/L), median [IQR]	56 [28–143]
hs-cTnT (ng/L), median [IQR]	18 [12–42]
Galectin-3 (μg/L), median [IQR]	10.5 [9.3–12.9]

*ACEi, angiotensin-converting enzyme inhibitor; ARB, angiotensin II receptor blocker; eGFR, estimated glomerular filtration rate; FEV1, forced expiratory volume in 1 second; FVC, forced vital capacity; hs-cTnT, high-sensitivity cardiac Troponin-T; LVEF, left ventricular ejection fraction; MRA, mineralocorticoid receptor antagonist; NT-proBNP, N-terminal pro-B-type natriuretic peptide*.

### Correlation of eGFR Assessed by Creatinine and Cystatin C With Kidney Function

**Figure 1:**
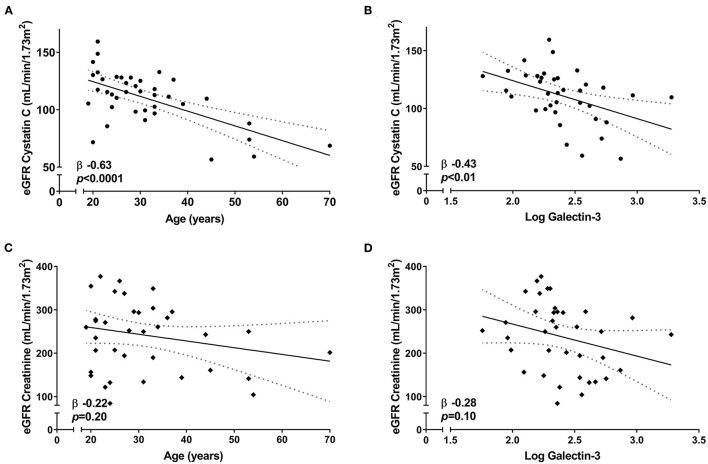
Correlation between eGFR and renal parameters among the 39 neuromuscular dystrophy patients in this study. Linear regression [95% CI] showing the correlation of Cystatin C-based eGFR with **(A)** age and **(B)** galectin-3 levels. Linear regression [95% CI] showing the correlation of creatinine-based eGFR with **(C)** age and **(D)** galectin-3 levels.

First, we determined that creatinine eGFR and Cystatin C eGFR were not associated with each other (β 0.28, *p* = 0.09). Second, we investigated whether age and galectin-3, which are both known parameters independently associated with kidney function (14–16), were associated with eGFR based on either creatinine or Cystatin C. Cystatin C-based eGFR was significantly associated with both age (β −0.63, *p* < 0.0001) and galectin-3 (β −0.43, *p* < 0.01) (Figures 1A,B), while creatinine-based eGFR did not show significant associations (β −0.22, *p* = 0.20 and β −0.28, *p* = 0.10, respectively) (Figures 1C,D).

### Sensitivity Analysis

Since the majority of our patient population consisted of Duchenne and Becker Muscular Dystrophy patients, that is particularly characterized by low muscle mass, we performed a sensitivity analysis in this patient group. We provide the clinical characteristics of this sub-group compared with the rest of the study population in Table 2. Duchenne and Becker patients were in higher need of respiratory care compared to other neuromuscular dystrophy patients, had a lower LVEF (41 vs. 53%, *p* = 0.002), higher NT-pro-BNP (110 vs. 31 ng/L, *p* < 0.001) and higher hs-TnT levels (31 vs. 14 ng/L, *p* = 0.008). 83% of the patients were treated with ACEi, ARBs, or MRAs. As expected, Duchenne and Becker patients had lower plasma creatinine levels (8 vs. 27 μmol/L, *p* = 0.003) and higher calculated creatinine-based eGFR (284 vs. 182 mL/min/1.73 m^2^, *p* < 0.001), while Cystatin C (0.81 vs. 0.86 mg/L, *p* = 0.26), and Cystatin C-based eGFR (114 vs. 105 mL/min/1.73 m^2^, *p* = 0.22) were not significantly different when compared to the rest of the study population.

**Table 2:** Baseline characteristics of Duchenne and becker muscular dystrophy patients and other neuromuscular dystrophy patients.

**Characteristics**	**Duchenne and Becker muscular**	**Other (*n* = 16)**	***p***
	**dystrophy (*n* = 23)**		
Age at evaluation (y), mean (SD)	30 (8)	33 (15)	0.61
Male sex, *n* (%)	23 (100)	9 (56)	** <0.001**
Body mass index (kg/m^2^), mean (SD)	23 (4)	22 (6)	0.48
**Respiratory status**			
FEV1 (L), mean (SD)	1.6 (1.3)	2.6 (1.5)	0.066
FVC (L), mean (SD)	1.8 (1.4)	2.8 (1.3)	0.051
No support, *n* (%)	9 (39)	12 (75)	**0.027**
Invasive ventilation, *n* (%)	6 (26)	3 (19)	0.59
Nocturnal support, *n* (%)	6 (26)	3 (19)	0.59
All day support, *n* (%)	8 (35)	1 (6)	**0.038**
**Ejection fraction (%), mean (SD)**	41 (13)	53 (4)	**0.002**
LVEF ≥50, *n* (%)	10 (43)	13 (81)	**0.018**
LVEF 40–49, *n* (%)	6 (26)	3 (19)	0.59
LVEF <40, *n* (%)	7 (30)	0 (0)	**0.015**
**Medication**, ***n*** **(%)**			
Corticosteroids	1 (4)	0 (0)	0.40
ACEi	18 (78)	0 (0)	** <0.001**
ARB	2 (9)	0 (0)	0.23
MRA	1 (4)	2 (13)	0.35
**Laboratory measurements**			
Cystatin C (mg/L), median [IQR]	0.81 [0.70–0.85]	0.86 [0.74–0.97]	0.26
Creatinine (umol/L), median [IQR]	8 [6–12]	27 [10–49]	**0.003**
Cystatin C-based eGFR (mL/min/1.73 m^2^), mean (SD)	114 (16)	105 (30)	0.22
Creatinine-based eGFR (mL/min/1.73 m^2^), mean (SD)	284 (60)	182 (68)	** <0.001**
NT-proBNP (ng/L), median [IQR]	110 [45–275]	31 [20–40]	** <0.001**
hs-cTnT (ng/L), median [IQR]	31 [13, 58]	14 [4, 20]	**0.008**
Galectin-3 (μg/L), median [IQR]	10.4 [9.2–12.7]	10.7 [9.6–13.5]	0.57

*ACEi, angiotensin-converting enzyme inhibitor; ARB, angiotensin II receptor blocker; eGFR, estimated glomerular filtration rate; FEV1, forced expiratory volume in 1 second; FVC, forced vital capacity; hs-cTnT, high-sensitivity cardiac Troponin-T; LVEF, left ventricular ejection fraction; MRA, mineralocorticoid receptor antagonist; NT-proBNP, N-terminal pro-B-type natriuretic peptide. Bold values denote statistical significance at the p < 0.05 level*.

**Figure 2:**
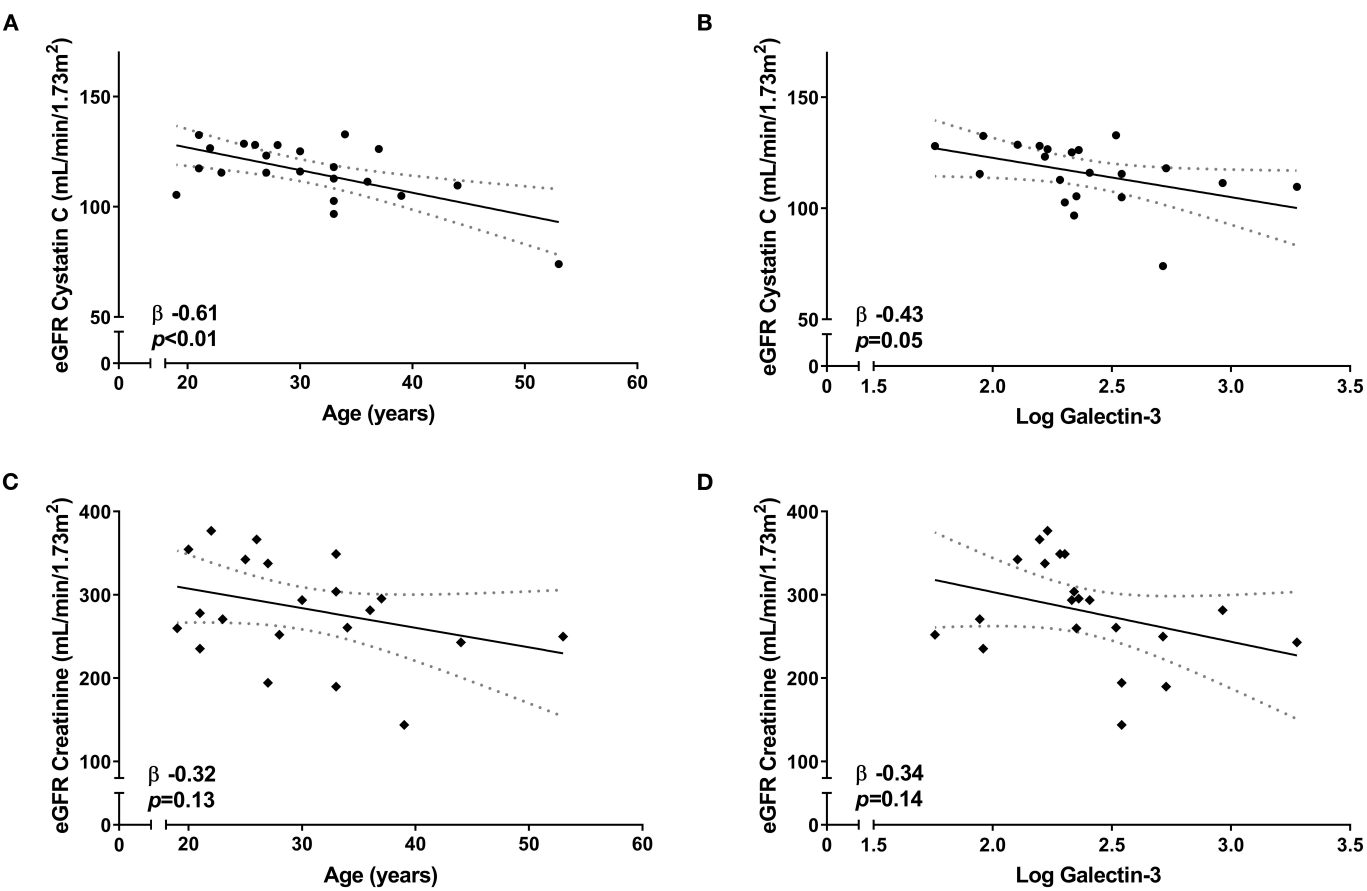
Correlation between eGFR and renal parameters in Duchenne and Becker muscular dystrophy patients. Linear regression [95% CI] showing the correlation of Cystatin C-based eGFR with **(A)** age and **(B)** galectin-3 levels. Linear regression [95% CI] showing the correlation of creatinine-based eGFR with **(C)** age and **(D)** galectin-3 levels.

A sensitivity analysis in the Duchenne and Becker population showed that Cystatin C-based eGFR was still associated with age (β −0.61, *p* < 0.01) and galectin-3 (β −0.43, *p* = 0.05) (Figures 2A,B), while creatinine-based eGFR was not (β −0.32, *p* = 0.13 and β −0.34, *p* = 0.14, respectively) (Figures 2C,D). Creatinine eGFR and Cystatin C eGFR were not associated with each other (β 0.02, *p* = 0.92).

## Discussion

This study demonstrates that kidney function calculated from plasma creatinine levels is unreliable in neuromuscular dystrophy patients and likely does not reflect the “real” kidney function. eGFR assessed by creatinine is not associated with known parameters of kidney function like age and galectin-3, and yields extreme estimates of renal function. Interestingly, eGFR assessed by Cystatin C seems to be a better alternative in this specific population, showing a strong association with both age and galectin-3; a decline in kidney function with age is expected in the general patient population (16, 17). The first longitudinal study from Rowe et al. (18) confirmed this observation. The same trend can be seen in neuromuscular dystrophy patients. A study of Braat et al. in pediatric and adolescent DMD patients also showed a clear association of measured GFR (i.e., ^51^Cr-EDTA) with age (7). As for galectin-3, it has been shown to be inversely related to kidney function (19). A study of Tang et al. showed that higher galectin-3 plasma levels were associated with renal dysfunction in patients with heart failure (20), and de Boer et al. reported that galectin-3 levels showed a close correlation with several parameters of renal function, including Cystatin C in the general population (21).

Furthermore, plasma creatinine levels were exceptionally low in our patient population when compared to values in healthy controls (22, 23), as can be expected due to the nature of the disease (8). Additionally, it is known that creatinine levels are highly dependent on age. Creatinine concentrations only rise to adult levels by about 15–17 years of age (23), while absolute renal function reaches adult values by the age of 2 (24). In adults, creatinine levels also steadily increase with age (25). By contrast, Cystatin C levels in our neuromuscular patient cohort were more comparable to levels in healthy individuals (8, 23, 26). Although creatinine levels are highly dependent on age, it has been shown that reference ranges for Cystatin C levels remain more similar during aging (23, 27). A study from Finney et al. in children and premature infants showed that Cystatin C levels are increased after birth, but fall to adult ranges from 1 to 3 years of age, mirroring measured GFR (i.e., ^51^Cr-EDTA clearance) (23). In adults, Cystatin C levels remain relatively constant and rise significantly only after 50 years of age (27).

Since Cystatin C levels do not change over time and during aging (26) and levels are independent of lean muscle mass (9, 10), our results indicate that serum Cystatin C level can be used for reliable evaluation of renal function in neuromuscular dystrophy patients. However, it has to be mentioned that a study from Knight et al. showed that older age was independently associated with higher serum Cystatin C levels in the PREVEND cohort of 8,592 healthy participants (28). This might indicate that the association between Cystatin C-based eGFR and age in our study is independent of renal function. Furthermore, Cystatin C levels might be influenced by corticosteroid use, drugs widely used as treatment in this patient population. While some studies mention normal levels of Cystatin C irrespective of corticosteroid therapy (8), others show slightly elevated levels, without decrease in renal function (29). Additionally, some common diseases, for instance cancer and thyroid disease, may also affect serum Cystatin C levels. In our study population, only 1 patient was treated with corticosteroids at baseline and 1 patient had a history of cancer. Although these confounders are unlikely to have major impact on our results, they need to be acknowledged when interpreting Cystatin C values properly, especially considering that some types of neuromuscular diseases are known to increase cancer risk (30).

While our patient population existed of a broad range of neuromuscular disorders, the majority was diagnosed with either Duchenne or Becker Muscular Dystrophy. For this reason, we performed a sensitivity analysis in this patient group. Cystatin C-based eGFR was associated with both age and galectin-3, while creatinine-based eGFR was not. These results are in accordance with previous studies in DMD patients, in which Cystatin C eGFR correlated better with renal function compared to creatinine eGFR (7, 8).

In this study, we only included patients that were evaluated for suspected or established cardiac involvement. In literature, it has been described that the prevalence rates of Duchenne and Becker Muscular Dystrophy are 3 and 2 per 100,000 people, respectively. Other neuromuscular disorders on the other hand, including myotonic dystrophy type 1, post-poliomyelitis syndrome (PPM), Charcot-Marie-Tooth Disease (CMT), hereditary neuropathy with pressure palsies (HNPP), and myasthenia gravis are 3 to 12 times more frequent in the general population (31). However, for DMD it is known that almost all patients will develop cardiac abnormalities over the age of 18 and that pre-clinical cardiac involvement can already be seen in a quarter of the population under the age of 6 (32). This makes cardiac disease the second cause of death. For this reason, DMD patients are often treated according to the adult HF guidelines, including treatment with ACEi, ARBs, and MRAs. The DMD Care Considerations Working Group even recommends ACE inhibitors as first-line therapy in DMD patients with left ventricular dysfunction (33). In our study, 56% of the patients were treated with either ACEi, ARBs, or MRAs. In the Duchenne and Becker population, this concerned 83% of the patients. Although these drugs have shown to improve left ventricular function in this specific patient group (34), they might also contribute to and worsen renal insufficiency (35).

For some other neuromuscular disorders, including CMT disease, cardiac involvement has only been described occasionally. Although a growing number of case reports describe arrhythmias (36), conduction disturbances and dilated cardiomyopathy, some investigators label them as fortuitous (37), or as a result of their medication (36). In a prospective study of 68 patients with CMT disease the frequency of cardiac abnormalities did not exceed the occurrence in the general population (38). As for myasthenia gravis, and other more common neuromuscular disorders, incidence and prevalence of cardiac involvement remains largely unknown (39). Additionally, overlapping symptoms (i.e., fatigue, dyspnea) and a reduced ability of physical activity might lead to under recognition of cardiac involvement in some neuromuscular disorders (39). By contrast, Myotonic Dystrophy type 1, the most common adult form of muscular dystrophy, affects ~ 1 in 8,000 people. It is estimated that cardiac abnormalities—mostly conduction disturbances or arrhythmia—appear in roughly 30–75% of the patient population (40). On the other hand, cardiomyopathy is much less common. A multicentre registry of 382 Myotonic Dystrophy type 1 patients showed some form of structural heart disease in <20% of the patients and HF could only be found in 1.8% (41). Based on this information, it is argumentative that the greater part of our study population suffers from Duchenne or Becker Muscular Dystrophy. Additionally, cardiac dysfunction, prolonged hypovolemia and cardiovascular medication might be main contributors of renal failure in patients with neuromuscular disorders. Kidney failure might therefore be less applicable in neuromuscular patients without cardiac involvement.

During the last decades, clinical treatment for neuromuscular patients has improved drastically, resulting in prolonged life expectancy (42). Therefore, monitoring and therapy are not only focused on respiratory and cardiovascular complications, but are expanded with focus on renal dysfunction. Although renal dysfunction has been described as rare in some types of neuromuscular diseases (43), other studies mention up to 82% of the patients suffering from renal failure to some extent (44). Low plasma creatinine levels due to muscle breakdown and commonly co-existence of cardiovascular complications, may result in misinterpretation of markers and overestimation of kidney function. This clearly shows there is a need for a useful and practical approach to determine and adequately monitor kidney function in patients with neuromuscular disorders.

## Limitations

There are a few limitations to this study. First, we did not perform GFR measurement by urinalysis—many patients are wheel chair bound and 24 h urine sampling present a very heavy burden to these patients. However, the lack of the gold standard for estimation of GFR makes this study observational and results should also be interpreted as such. Second, our patient cohort consisted of only 39 patients suffering from a broad range of neuromuscular disorders. The usefulness of Cystatin C in determination of kidney function might differ between several patient groups.

## Conclusion

Our data indicate that the use of creatinine to monitor renal function is severely limited in patients with neuromuscular disorders. Cystatin C may be a useful and minimally invasive biomarker for determination of renal function, particularly suitable in Duchenne or Becker Muscular Dystrophy patients, but certainly also applicable for other neuromuscular patients. Since neuromuscular disorders comprise a broad range of conditions that impair the functioning of the muscles (i.e., respiratory, circulatory, and renal failure), a variety of medical specialists evaluate neuromuscular patients and prescribe medication, potentially contributing to renal insufficiency. Therefore, we advocate the use of Cystatin C to assess kidney function in this particular patient population.

## Data Availability Statement

The raw data supporting the conclusions of this article will be made available by the authors, without undue reservation.

## Ethics Statement

Ethical review and approval was not required for the study on human participants in accordance with the local legislation and institutional requirements. Written informed consent for participation was not required for this study in accordance with the national legislation and the institutional requirements.

## Author Contributions

ES, WM, and RdB participated to the conceptualization and design of the study, data analysis, and/or interpretation of data. JK-R, JD, JN, HM, WM, and RdB contributed to data collection, and performed a critical and intellectual revision of the manuscript. ES contributed to acquisition of data and drafting the manuscript. RdB contributed to funding for publication. All authors participated sufficiently to the manuscript and approved the submitted version.

## Funding

This work was supported by grants from the Dutch Heart Foundation (CVON SHE-PREDICTS-HF, grant 2017-21; CVON RED-CVD, grant 2017-11; CVON PREDICT2, grant 2018-30; and CVON DOUBLE DOSE, grant 2020B005), by a grant from the leDucq Foundation (Cure PhosphoLambaN induced Cardiomyopathy (Cure-PLaN), and by a grant from the European Research Council (ERC CoG 818715, SECRETE-HF). Dr. Meijers is supported by the Mandema-Stipendium of the Junior Scientific Masterclass 2020-10, University Medical Center Groningen.

## Conflict of Interest

The UMCG, which employs the authors, has received research grants and/or fees from AstraZeneca, Abbott, Boehringer Ingelheim, Cardior Pharmaceuticals Gmbh, Ionis Pharmaceuticals, Inc., Novo Nordisk, and Roche. RdB received speaker fees from Abbott, AstraZeneca, Bayer, Novartis, and Roche.

## Publisher's Note

All claims expressed in this article are solely those of the authors and do not necessarily represent those of their affiliated organizations, or those of the publisher, the editors and the reviewers. Any product that may be evaluated in this article, or claim that may be made by its manufacturer, is not guaranteed or endorsed by the publisher.

## Abbreviations

ACEi: angiotensin-converting enzyme inhibitor
ARB: angiotensin II receptor blocker
CKD-EPI: Chronic Kidney Disease Epidemiology Collaboration
CMT: Charcot-Marie-Tooth Disease
DMD: Duchenne Muscular Dystrophy
eGFR: estimated glomerular filtration rate
FEV1: forced expiratory volume in 1 second
FVC: forced vital capacity
HF: heart failure
HNPP: hereditary neuropathy with pressure palsies
LVEF: left ventricular ejection fraction
PPM: poliomyelitis syndrome
MRA: mineralocorticoid receptor antagonist
NT-proBNP: N-terminal B-type natriuretic peptide
hs-TnT: high-sensitivity Troponin T.
